# London Hospital

**Published:** 1846-03

**Authors:** 


LONDON HOSPITAL.

*Hydrocele.—Difficult Diagnosis.*—Henry S——, a tall man, aged forty-three, applied at the hospital in August, on account of a large swelling of the scrotum on the right side. The swelling was of a pyriform shape, and fluctuated, but by no means distinctly. It extended as high up as the inguinal canal, which was full and dilated. The testicle could not be felt, but at the back of the tumour there was an induration which indicated its site, the usual pain being experienced on pressure there. The tumour received an impulse on coughing—an impulse as distinct as is usually felt in scrotal hernia. On examining the tumour in the ordinary way, no transparency could be detected; but on placing the patient in a room from which daylight was carefully excluded, and examining the swelling through a tube, formed with a roll of writing-paper, a lighted candle being placed at the opposite side, transparency could be clearly recognised. The patient stated that he had been subject to a swelling of the scrotum as long as he could remember. Mr. Curling gave his opinion that this was a case of hydrocele, the fluid being contained in a thickened sac, and an impulse on coughing being communicated to the swelling, in consequence of the sac extending as high up as the internal abdominal ring. He stated that the sac was of uneven thickness, and that, at its lowest part, there was a kind of pouch, the sac being thinner at this part, and the transparency, consequently, more evident. As a matter of precaution, he carefully cut down to the sac at this part with a scalpel, and made a small opening in it. About twenty-seven ounces of fluid, of a darker colour than usual, and thickly loaded with cholesterine, escaped. The testis was found rather large, and the epididymis and vas deferens a good deal thickened. Inflammation of the tunica vaginalis followed the operation, and at one time there was a prospect of the hydrocele being cured by it. But after some weeks, the fluid appeared to be returning, and the patient has since discontinued to attend.

*Encysted Hydrocele of the Epididymus, combined with Varicocele, consequent on an Injury.—Difficult Diagnosis.—Relieved by Puncture and the Lever Truss.*—Morris L——, aged forty-two, a stout German Jew, came under the care of Mr. Curling, as an out patient, in consequence of a swelling of the left testis. He attributed the origin of his complaint to a blow received six months previously. It caused considerable pain and swelling at the time, and he was admitted into the hospital, where he remained several weeks, and was then discharged. There was a soft doughy swelling at the upper and back part of the testis, and connected with it, and extending upwards to the inguinal canal, a large number of varicose veins; but the swelling was obviously something more than a tumour occasioned by dilatation of the spermatic veins. It felt irregular, and communicated to the fingers an indistinct sense of fluctuation; but on careful and repeated examination in a darkened room, not the slightest transparency could be detected. The part felt heavy and uncomfortable, and, at times, quite painful, so that the patient could not continue his usual occupation. Various external applications were used, such as mercurial and iodine ointments, and iodine paint, but without relieving the pain, or causing any reduction of the swelling, which remained for some weeks in a stationary condition. Suspecting that the swelling might partly consist of an encysted hydrocele of the epididymis, the transparency of which was completely obscured by the varicose veins distributed around the tumour, Mr. Curling introduced a grooved needle into the upper part, when the escape of a few drops of a clear pellucid fluid revealed the true nature of the case. An adjoining but separate and smaller cyst was afterward punctured in a similar manner. In a few days afterwards, the swelling had subsided a good deal, and the part was freer from pain, but it still felt heavy and uneasy at times. In about three weeks the swelling had increased to its former size, and it was again relieved in the same manner as before. Pressure at the external abdominal ring was found to relieve the sense of weight, and the uneasiness in the testis. The moc-main lever truss was consequently applied, and the patient derived so much benefit from wearing it, that he shortly afterwards desired to be discharged.—*London Lancet.*

